# The double-edged sword of daptomycin: a case of daptomycin-induced eosinophilic pneumonia

**DOI:** 10.1093/omcr/omag146

**Published:** 2026-08-11

**Authors:** Mohammed Najdat Seijari, Fayaz Khan, Taraneh Zamani, Shahla Mallick, Mohammad Nasser Affas, Stephen Beerman

**Affiliations:** Department of Internal Medicine, Good Samaritan Hospital, TriHealth, 375 Dixmyth Ave, Mount Auburn neighborhood, Cincinnati, OH 45220, United States; Department of Internal Medicine, Good Samaritan Hospital, TriHealth, 375 Dixmyth Ave, Mount Auburn neighborhood, Cincinnati, OH 45220, United States; Department of Internal Medicine, Good Samaritan Hospital, TriHealth, 375 Dixmyth Ave, Mount Auburn neighborhood, Cincinnati, OH 45220, United States; Department of Pulmonary and Critical Care Medicine, Good Samaritan Hospital, TriHealth, 375 Dixmyth Ave, Mount Auburn, Cincinnati, OH 45220, United States; Department of Internal Medicine, Hamad Medical Corporation, Al Rayyan Road, Hamad Medical City, Doha, Qatar; Department of Internal Medicine, Good Samaritan Hospital, TriHealth, 375 Dixmyth Ave, Mount Auburn neighborhood, Cincinnati, OH 45220, United States

**Keywords:** Daptomycin, Eosinophilic pneumonia, Antibiotic toxicity, Drug-induced lung disease

## Abstract

Daptomycin is an effective antibiotic for resistant gram-positive infections, including MRSA and VRE, but can rarely cause daptomycin-induced eosinophilic pneumonia (DIEP). We present a 73-year-old woman treated for prosthetic joint infection who developed acute hypoxemic respiratory failure and peripheral eosinophilia after 21 days of daptomycin therapy. Chest imaging showed bilateral upper lobe consolidations. Symptoms resolved rapidly after stopping daptomycin, without corticosteroid use. This case highlights the need for early recognition of DIEP in patients presenting with new respiratory symptoms and eosinophilia during daptomycin therapy, enabling timely discontinuation and reducing potential morbidity.

## Introduction

Daptomycin, a lipopeptide antibiotic, is widely used for infections caused by resistant gram-positive bacteria, including MRSA and VRE. Though generally well tolerated, it can rarely induce eosinophilic pneumonia—a potentially life-threatening hypersensitivity reaction. Awareness of this complication is crucial for timely diagnosis and management.

## Case presentation

A 73-year-old woman with a prosthetic left knee joint infection was receiving intravenous daptomycin (6 mg/kg/day) and cefepime via a peripherally inserted central catheter. After 21 days of therapy, she presented from a skilled nursing facility with low-grade fever, chills, progressive dyspnea, and fatigue. Initial oxygen saturation was 80% on room air, necessitating supplemental oxygen.

Septic workup, including multiple blood cultures, was negative. Peripheral eosinophilia was noted (700/μl, peaking at 1100/μl). Chest computed tomography (CT) revealed bilateral upper lobe consolidations with ground-glass opacities (Fig. 2). DIEP was suspected based on the clinical picture and temporal correlation with daptomycin therapy.

**Figure 1:**
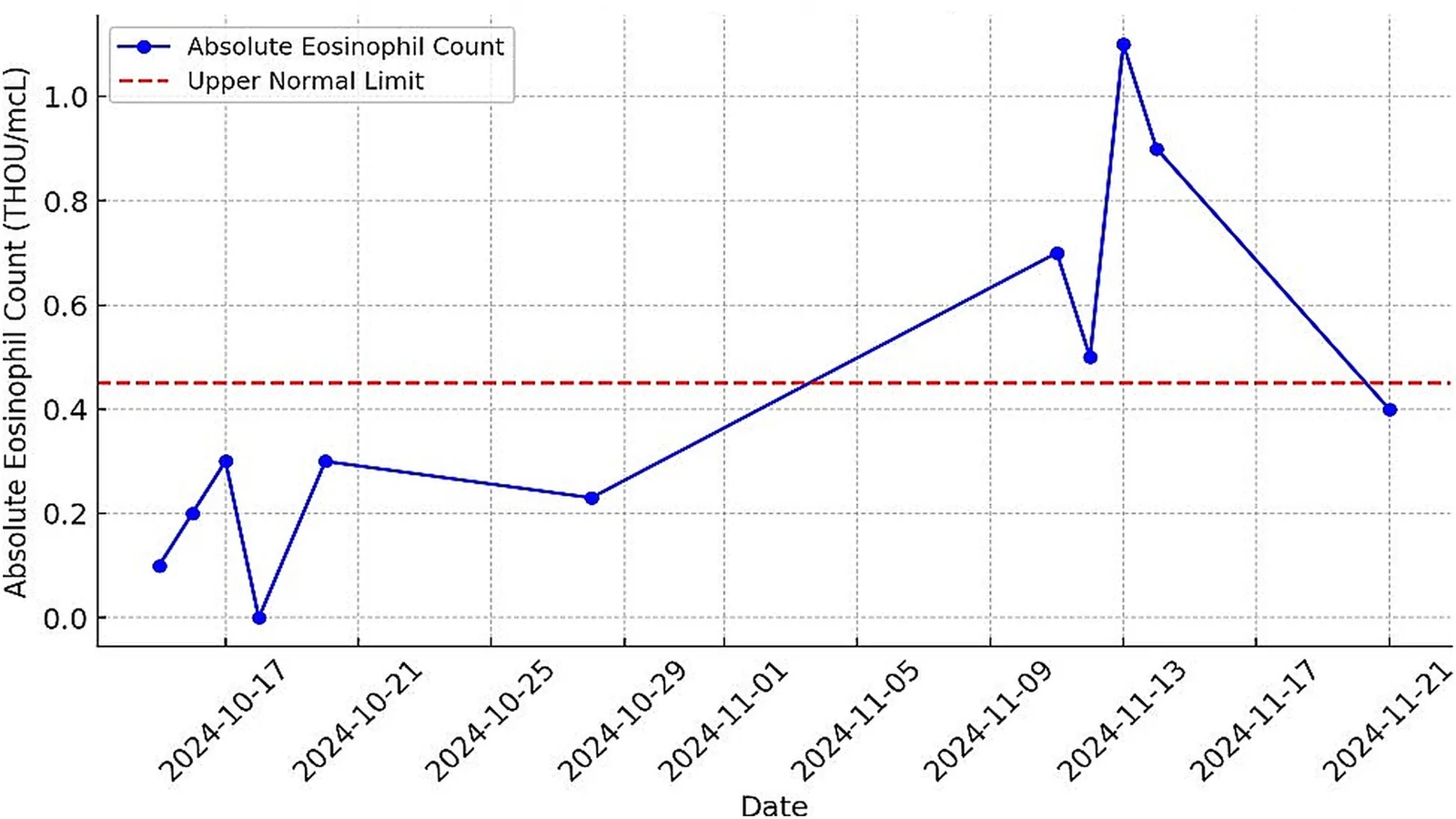
Eosinophil trend chart showing the uptrend of eosinophils after starting Daptomycin and rapid improvement after discontinuing the medication (medication was stopped 11/13/2024).

Daptomycin was discontinued and replaced with linezolid. Evaluation for alternative causes—including parasitic and fungal infections, autoimmune disorders, and vasculitides—was unremarkable (Table 1). Notably, the patient’s eosinophil count began to decline spontaneously (Fig. 1), and her respiratory symptoms resolved within 72 hours. She was weaned off oxygen, and inflammatory markers normalized without corticosteroid therapy. Bronchoscopy was deferred due to clinical improvement. A follow-up CT scan at six weeks showed complete resolution of prior abnormalities (Fig. 3). A diagnosis of probable DIEP was made.

**Table 1:** Laboratory and Immunologic Studies.

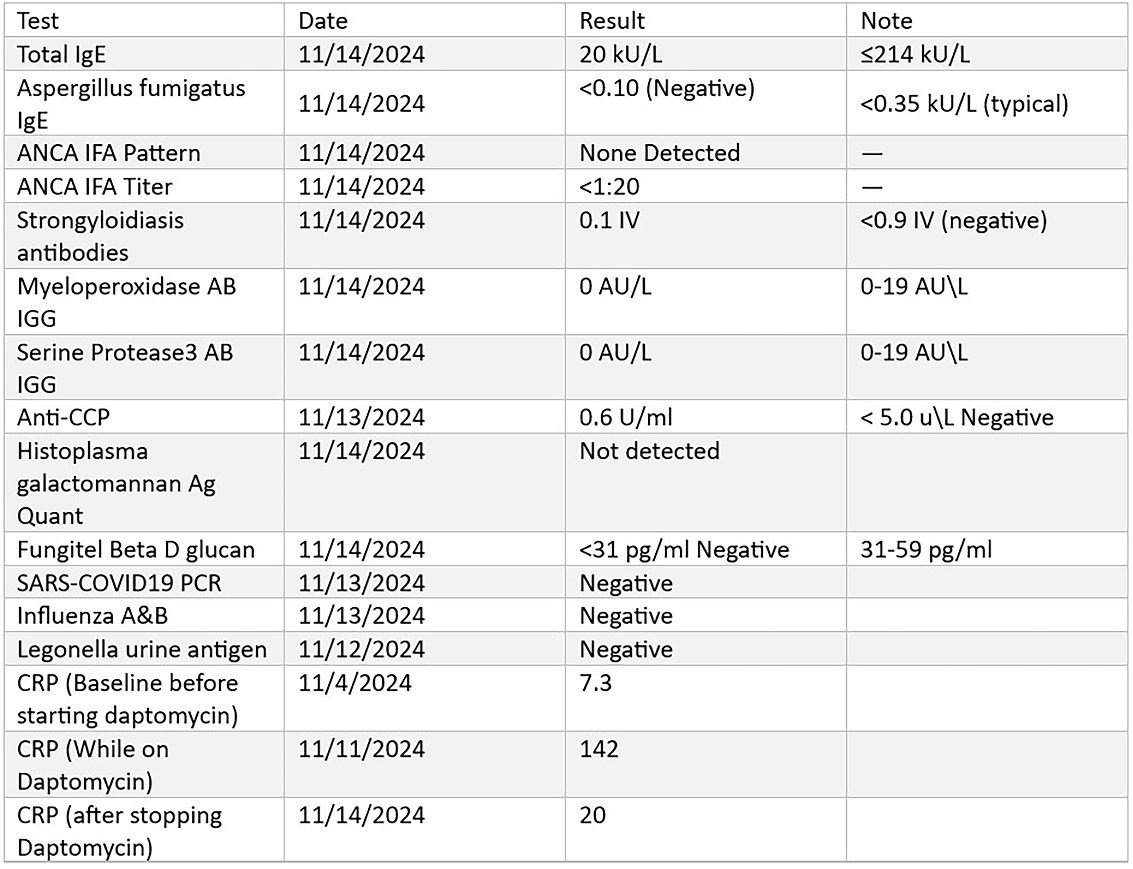

**Figure 2:**
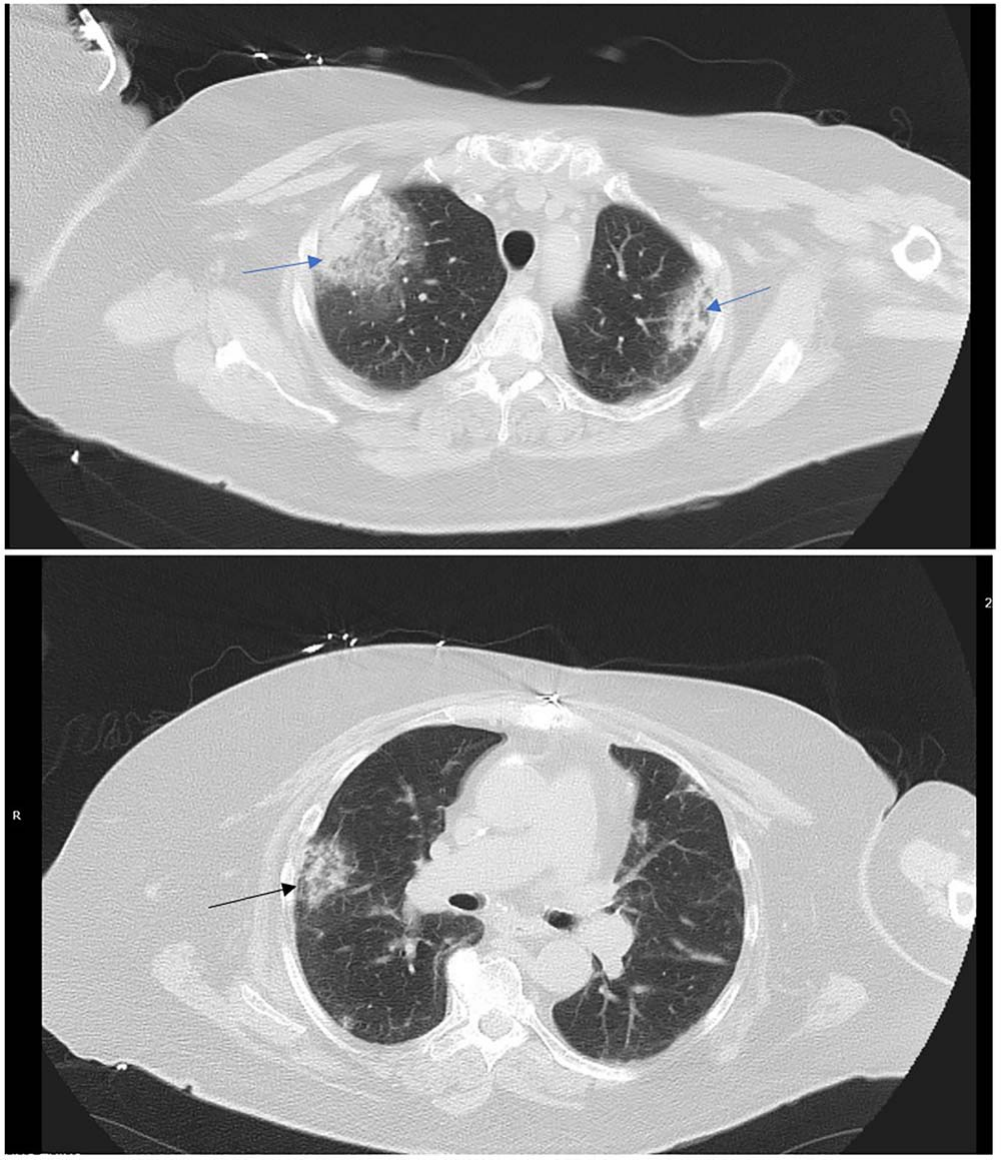
CT Chest without contrast (Before discontinuation of Daptomycin) showing new consolidative changes in both upper lobes, mainly the right upper lobe (blue arrows), with ground glass opacities extending to the right middle lobe (black arrow).

**Figure 3:**
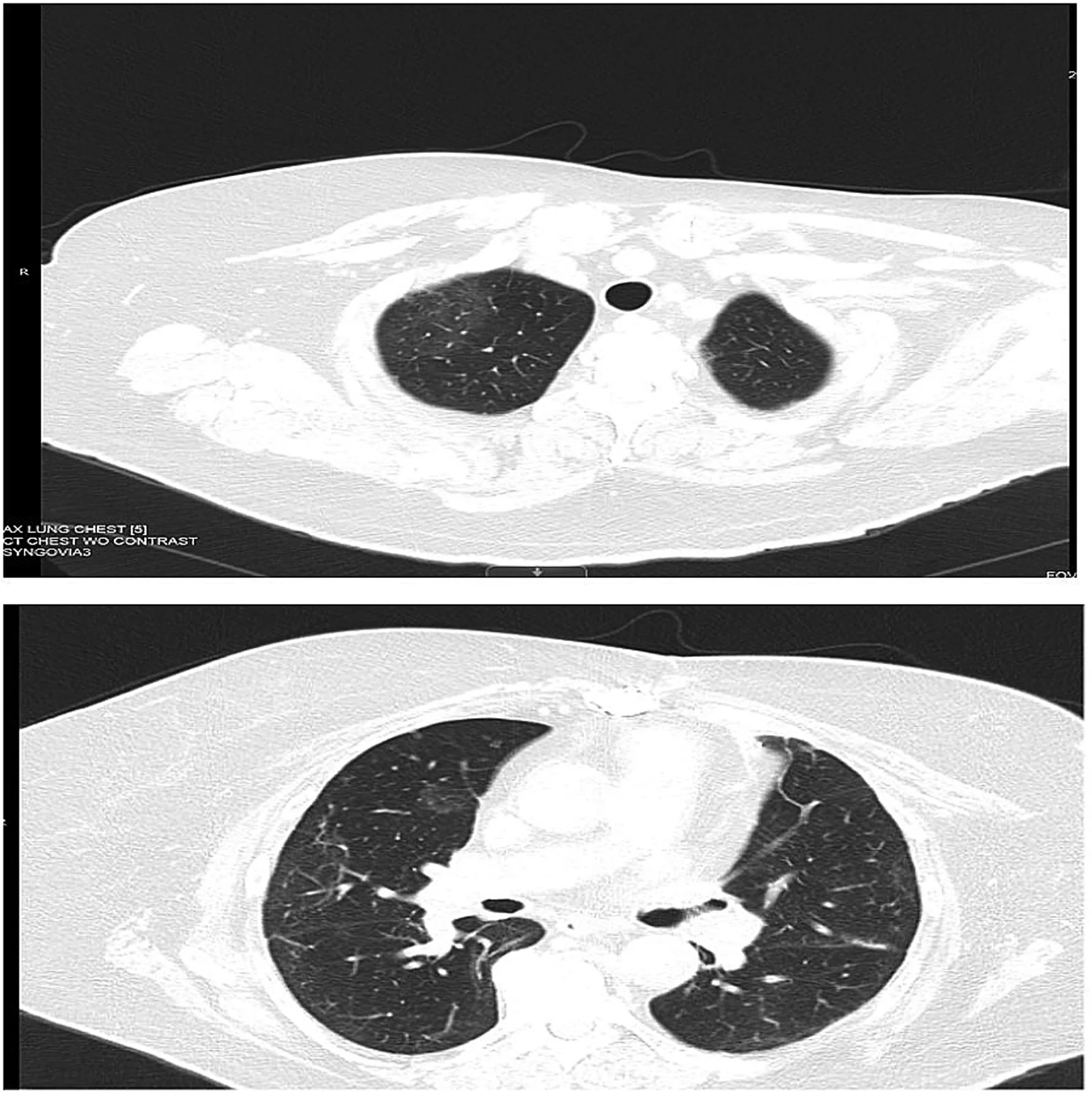
CT without contrast, 6 weeks after discontinuing Daptomycin, showing resolution of prior changes.

## Discussion

Daptomycin is a lipopeptide antibiotic that works by calcium-dependent insertion into bacterial membranes, causing potassium efflux and membrane depolarization, ultimately leading to cell death [1]. It is commonly used to treat various systemic infections where MRSA is a potential cause, including prosthetic joint infections [2].

While daptomycin presents an advantageous alternative to vancomycin due to its lower risk of severe side effects such as nephrotoxicity and its easier-to-manage dosing [3], it is not without adverse effects.

One of the rare but potentially severe adverse events is daptomycin-induced eosinophilic pneumonia (DIEP), which typically presents with respiratory symptoms and eosinophilia, as with this case. The precise mechanism behind DIEP is not fully understood. It is hypothesized that daptomycin binds to pulmonary surfactant, disrupting alveolar homeostasis and directly injuring alveolar epithelial cells. This injury triggers a hypersensitivity-type immune reaction characterized by the release of eosinophil chemoattractants such as interleukin-5 (IL-5) and serotonin, ultimately leading to eosinophilic infiltration and interstitial lung inflammation [4]. Notably, daptomycin’s inactivation by lung surfactant might explain the drug’s lack of efficacy in pulmonary infections while paradoxically being implicated in lung toxicity [5].

Several risk factors have been linked to DIEP, including age over 70 years, extended exposure to daptomycin exceeding 14 days, and a total cumulative dose of more than 10–15 grams. Our patient exhibited these risk factors [6].

The American Thoracic Society (ATS) classifies DIEP into definite, probable, possible, and unlikely categories. The six criteria for a definite diagnosis include: simultaneous daptomycin exposure, fever, dyspnea requiring more oxygen or mechanical ventilation, new chest infiltrates, bronchoalveolar lavage (BAL) showing > 25% eosinophils, and improvement after daptomycin withdrawal [7]. Probable cases may have < 25% eosinophils on BAL or only peripheral eosinophilia but meet the other criteria. Our patient met five of the six criteria, classifying it as a probable case.

The diagnosis of DIEP is challenging due to the restrictive diagnostic criteria set by the ATS. Although BAL with eosinophil count is considered essential for a definitive diagnosis, the procedure was not performed in our case due to the patient’s rapid improvement following daptomycin cessation. BAL is performed in approximately 62% of DIEP cases, and even when it is done, eosinophil counts are significantly elevated in fewer than half of the cases. This may be because daptomycin is typically withdrawn quickly once DIEP is suspected, potentially reducing eosinophil counts by the time the procedure is carried out, resulting in falsely low levels [8]. We wonder whether other parameters that could be easily measured, such as IL-5 or peripheral eosinophils, could serve as alternatives in the future.

The presentation of acute respiratory failure with peripheral eosinophilia and new pulmonary imaging findings could suggest various etiologies, including drug-induced causes, occupational and environmental exposures (e.g., hypersensitivity pneumonitis), infectious causes (e.g., parasites, fungi), and vasculitides (e.g., eosinophilic granulomatosis with polyangiitis). Additionally, DIEP may mimic more common conditions such as infectious pneumonia, COVID-19, pulmonary edema, or heart failure [8]. The workup was negative for all of these conditions (Table 1).

Definitive management of DIEP involves immediate discontinuation of the offending agent, which typically leads to clinical improvement. Steroids decrease eosinophil counts through multiple mechanisms, such as inhibiting eosinophil generation and activation while also inducing apoptosis [9]. Currently, there are no established guidelines regarding the indication, timing, or dosing of corticosteroid therapy. However, corticosteroids are commonly considered in severe cases, such as those requiring intubation, or when respiratory function does not improve rapidly following drug withdrawal. Supportive care, including oxygen therapy, should be provided as needed [10]. Our institution uses a dose of 1 mg/kg/day with a taper over 1–2 weeks. In our patient, steroids were not used due to the rapid improvement after daptomycin cessation. In general, improvement in DIEP is usually seen within 24 hours to 1 week after stopping the offending drug [5].

Although prognosis is generally favorable, severe and occasionally fatal cases have been reported despite corticosteroid therapy, with mortality occurring in 2 cases (2.7%) in a systematic review by Gidari et al. (2024) [8], physicians should remain vigilant as it can progress to fatal outcomes due to severe hypoxic respiratory failure [11].

While it might be tempting to consider rechallenging patients with a lower dose of daptomycin, we believe this practice should be avoided due to the high risk of recurrence. Immune cells may already be sensitized following the initial episode, and DIEP is known to be time- rather than dose-dependent. In a review by Kim et al., among 35 DIEP cases, three patients were rechallenged with daptomycin, and all three experienced a recurrence of symptoms and failure of the rechallenge [7]. Furthermore, one of the takeaways from this case could be to consider monitoring CBC while patients are on an extended course of daptomycin.

## Conclusion

DIEP is a rare but serious complication of daptomycin therapy. High clinical suspicion is warranted in patients with new pulmonary symptoms and eosinophilia. Prompt discontinuation of daptomycin typically leads to rapid improvement and may obviate the need for corticosteroids. Clinicians should avoid rechallenge and consider routine blood monitoring in patients on extended daptomycin courses. This case further emphasizes that DIEP can be reliably suspected on clinical grounds even without BAL confirmation, particularly when there is characteristic improvement after drug withdrawal, helping to prevent delayed diagnosis and unnecessary corticosteroid exposure.

## Limitations

This case has several limitations. First, bronchoalveolar lavage was not performed due to rapid clinical improvement, and therefore the diagnosis remains probable rather than definitive according to ATS criteria. Second, as a single case report, the findings are not generalizable. Although an extensive diagnostic workup was negative, alternative etiologies cannot be completely excluded. Third, causality is supported by temporal association and resolution after daptomycin withdrawal; however, lack of rechallenge limits definitive confirmation. Additionally, the absence of corticosteroid use, while clinically informative, limits comparison with most reported DIEP cases. Radiologic findings were non-specific, and no cytokine or biomarker analyses were performed to further support the proposed pathophysiological mechanism. Finally, follow-up duration was limited, and long-term outcomes remain unknown.
